# Laryngeal mask use during neonatal resuscitation at birth: A United States-based survey of neonatal resuscitation program providers and instructors

**DOI:** 10.1016/j.resplu.2023.100515

**Published:** 2023-11-30

**Authors:** Elizabeth E. Foglia, Birju A. Shah, Lise DeShea, Kathryn Lander, Beena D. Kamath-Rayne, Heidi M. Herrick, Jeanette Zaichkin, Sura Lee, Christopher Bonafide, Clara Song, Gene Hallford, Henry C. Lee, Vishal Kapadia, Tina Leone, Justin Josephsen, Arun Gupta, Marya L. Strand, William H. Beasley, Edgardo Szyld, for the American Academy of Pediatrics Delivery Room Intervention, Evaluation DRIVE Network

**Affiliations:** a Department of Pediatrics, Children’s Hospital of Philadelphia, Philadelphia, PA, United States; b Department of Pediatrics, University of Oklahoma Health Sciences Center, Oklahoma City, OK, United States; c Global Child Health and Life Support, American Academy of Pediatrics, Itasca, IL, United States; d Positive Pressure, PLLC, Shelton, WA, United States; e Southern California Permanente Medical Group, Anaheim, CA, United States; f Division of Neonatology, University of California San Diego School of Medicine, La Jolla, CA, United States; g Division of Neonatology, Department of Pediatrics, UT Southwestern, Dallas, TX, United States; h Division of Neonatology, Columbia University Vagelos College of Physicians and Surgeons, New York, NY, United States; i Division of Neonatology, Saint Louis University School of Medicine, St. Louis, MO, United States; j Department of Pediatrics, Stanford University School of Medicine, Palo Alto, CA, United States

**Keywords:** Newborn, Resuscitation, Laryngeal Mask, Survey

## Abstract

**Aim:**

Neonatal resuscitation guidelines promote the laryngeal mask (LM) interface for positive pressure ventilation (PPV), but little is known about how the LM is used among Neonatal Resuscitation Program (NRP) Providers and Instructors. The study aim was to characterize the training, experience, confidence, and perspectives of NRP Providers and Instructors regarding LM use during neonatal resuscitation at birth.

**Methods:**

A voluntary anonymous survey was emailed to all NRP Providers and Instructors. Survey items addressed training, experience, confidence, and barriers for LM use during resuscitation. Associations between respondent characteristics and outcomes of both LM experience and confidence were assessed using logistic regression.

**Results:**

Between 11/7/22–12/12/22, there were 5,809 survey respondents: 68% were NRP Providers, 55% were nurses, and 87% worked in a hospital setting. Of these, 12% had ever placed a LM during newborn resuscitation, and 25% felt very or completely confident using a LM. In logistic regression, clinical or simulated hands-on training, NRP Instructor role, professional role, and practice setting were all associated with both LM experience and confidence.

The three most frequently identified barriers to LM use were insufficient experience (46%), preference for other interfaces (25%), and failure to consider the LM during resuscitation (21%). One-third (33%) reported that LMs are not available where they resuscitate newborns.

**Conclusion:**

Few NRP providers and instructors use the LM during neonatal resuscitation. Strategies to increase LM use include hands-on clinical training, outreach promoting the advantages of the LM compared to other interfaces, and improving availability of the LM in delivery settings.

## Introduction

Up to 10% of all newborns require resuscitation to breathe immediately after birth.^1^ Positive pressure ventilation (PPV) is the most important intervention during neonatal resuscitation.^1^, ^2^ PPV is typically performed with a facemask, but mask leak and airway obstruction are common obstacles to effective ventilation. American Heart Association/American Academy of Pediatrics Neonatal Resuscitation Guidelines recommend “ventilation corrective steps” to troubleshoot impediments.^3^ However, these steps are variably performed, can worsen ventilation quality, and may delay advanced airway placement, prolonging bradycardia and hypoxia.^4^

The endotracheal tube is the most common alternative airway used during neonatal resuscitation. However, endotracheal intubation requires advanced technical skills and carries significant procedural risks.^5^ A laryngeal mask (LM), or supraglottic airway, is an alternative airway that is widely available and promoted in neonatal resuscitation guidelines.^3^ Despite this, little is known about LM use during neonatal resuscitation in high-resource settings. A single-center study in the United States (US) identified barriers to LM use including limited experience, insufficient training, endotracheal tube preference, and lack of awareness.^6^

Little is known about Neonatal Resuscitation Program (NRP) Providers’ use and perceptions for the LM during neonatal resuscitation. We conducted this survey to characterize current experience, use, perceived barriers, and implementation readiness for the LM across a contemporary cohort of NRP Providers and Instructors.

## Methods

An invitation to participate in the survey was disseminated from the American Academy of Pediatrics to email addresses of 425,762 NRP Providers and Instructors on the NRP listserv. Most listserv members are US-based. Interested respondents accessed an embedded link to an anonymous electronic survey between 11/7/22 and 12/12/22. Study data were collected and managed using REDCap (Research Electronic Data Capture) electronic data capture tools hosted at University of Oklahoma Health Sciences Center.^7^, ^8^ Two reminder emails were sent with two weeks between emails, and the survey was closed one week after the final email reminder. The American Academy of Pediatrics and the University of Oklahoma Health Sciences Center Institutional Review Boards reviewed this study and deemed it exempt from oversight. Written informed consent was waived; the invitation specified that participation was voluntary and that participation in the survey implied consent.

The survey (supplemental material) included questions adapted from a previous single center study.^6^ Consistent with the NRP terminology, we used the term “laryngeal mask.”^9^ Survey items addressed demographic characteristics, training, experience, and confidence using a LM during resuscitation. The survey prompted respondents to identify up to three barriers to LM use from a prespecified list. Additional questions pertained to LM availability and respondents’ perceptions around appropriate timing of the LM use during neonatal resuscitation. Three items assessed LM implementation acceptability, appropriateness, and feasibility on a 5-point Likert scale, using prompts adapted from a validated implementation outcome assessment tool.^10^ Likert scale responses of “agree” and “completely agree” were consolidated as “agree” for reporting purposes.

The primary outcomes of interest were previous LM use during neonatal resuscitation (yes/no) and confidence using the LM during neonatal resuscitation, measured on a Likert scale from “not at all confident” to “completely confident.” For analysis and reporting purposes, responses of “very confident” and “completely confident” were consolidated as “confident,” with all other responses being combined as “not confident.” Responses for each outcome were summarized based on respondent subgroups related to role, practice setting, and LM training. Generalized linear models with a binomial outcome and a logistic linking function were run in R, version 4.2.3, to analyze the association between respondent characteristics and the two primary outcomes. The logistic model for each outcome included 7 predictors: professional role; current NRP role (Instructor or Provider); primary work setting; number of times participating in newborn resuscitation in the past six months; and binary indicators of having completed didactic, simulated, and clinical training with a LM. A p-value of 0.05 was considered statistically significant.

## Results

Responses were received from 5,809 (1.4%) of the listserv members (Table 1). Only 12% of respondents had ever placed a LM during newborn resuscitation, and 25% felt very or completely confident using a LM.

**Table 1:** Respondent Characteristics.

Characteristic (*N* = 5,809)	*n* (%)^1^
Current NRP Role (*n* = 5,556)^2^	
Instructor	1,801 (32%)
Provider	3,755 (68%)
Professional Role (*n* = 5,487)^2^	
Nurse	3,041 (55%)
Respiratory Therapist	894 (16%)
Physician	784 (14%)
Neonatology	395/784 (50%)
General Pediatrics/Hospitalist	259/784 (33%)
Family Medicine	89/784 (11%)
Emergency Medicine	16/784 (2%)
Other	39/784 (5%)
Advanced Practice Provider	463 (8%)
Certified Nurse Midwife	132 (2%)
First Responder (Emergency Medical Technician, Paramedic)	131 (2%)
Other	42 (1%)
Number of Times Neonatal Resuscitation Performed in Last 6 Months (*n* = 5,728)^2^	
Never	1,613 (28%)
1–4 times	2,353 (41%)
5–10 times	785 (14%)
More than 10 times	977 (17%)
Training in Laryngeal Mask^3^ (*n* = 5,730)^2^	
Hands-on, clinical	414 (7%)
Hands-on, simulation	3,940 (69%)
Didactic	2,232 (39%)
Most Frequent Practice Setting (*n* = 5,695)^2^	
Hospital	4,966 (87%)
Birth center	279 (5%)
Patient home	187 (3%)
Pre-hospital	263 (5%)
Hospital Details	
Teaching hospital (*n* = 4,929)^2^	2,558 (52%)
Neonatal care (*n* = 4,924)^2^	
I	720 (15%)
II	1,085 (22%)
III	1,774 (36%)
IV	808 (16%)
None	211 (4%)
Don’t know	326 (7%)

1 Group frequencies may not sum to the total N because of missing values. Percentages may not sum to 100 because of rounding.

2 Number of valid observations for this variable.

3 Training types are not mutually exclusive, so percentages will not sum to 100.

Responses for each of these outcomes based on respondent subgroups are shown in Table 2. Clinical or simulated hands-on training, NRP Instructor role, newborn resuscitation experience, and practice setting were all associated with both LM use and confidence. There were significant differences in both outcomes (previous LM use and confidence) based on professional role (Table 3).

**Table 2:** Experience and confidence placing a laryngeal mask during neonatal resuscitation, based on respondent subgroups.

Respondent Characteristic	Experience placing laryngeal mask, *n*/*N* (%)	Confidence placing laryngeal mask^1^^,^ *n*/*N* (%)
NRP Role		
Provider	368/3746 (10%)	771/3733 (21%)
Instructor	287/1787 (16%)	643/1789 (36%)
Professional Role		
Advanced Practice Provider	75/462 (16%)	158/460 (34%)
Certified Nurse Midwife	17/132 (13%)	28/131 (21%)
First Responder (EMT, Paramedic)	24/130 (18%)	74/129 (57%)
Nurse	206/3026 (7%)	456/3027 (15%)
Physician	158/783 (20%)	276/779 (35%)
Respiratory Therapist	136/888 (15%)	332/885 (38%)
Other	6/42 (14%)	17/42 (40%)
Resuscitation Experience in last 6 months		
0	105/1608 (7%)	330/1604 (21%)
1–4	278/2343 (12%)	551/2339 (24%)
5–10	99/781 (13%)	205/781 (26%)
>10	186/975 (19%)	346/974 (36%)
Practice Setting		
Non-hospital	127/726 (17%)	264/726 (36%)
Hospital, no/unknown neonatal care	40/534 (7%)	114/529 (22%)
Hospital, level I/II neonatal care	202/1795 (11%)	366/1793 (20%)
Hospital, level III/IV neonatal care	298/2575 (11%)	667/2572 (26%)
Didactic training in laryngeal mask		
Yes	304/2227 (14%)	672/2221 (30%)
No	365/3503 (10%)	766/3500 (22%)
Simulation training in laryngeal mask		
Yes	566/3927 (14%)	1255/3923 (32%)
No	103/1803 (6%)	183/1798 (10%)
Clinical training in laryngeal mask		
Yes	192/414 (46%)	253/413 (61%)
No	477/5316 (9%)	1185/5308 (22%)

Abbreviations: EMT: Emergency Medical Technician; NRP: Neonatal Resuscitation Program.

1 Confidence includes respondents who answered “very” or “completely” confident.

**Table 3:** Multivariable analysis of factors associated with experience and confidence placing a laryngeal mask during neonatal resuscitation.

Characteristic	Experience Placing Laryngeal Mask		Confidence Placing Laryngeal Mask^1^	
	aOR	95% CI	aOR	95% CI
NRP Instructor (Ref: NRP Provider)	1.71	1.40, 2.09	2.64	2.27, 3.08
Professional Role (Ref: Physician)				
Advanced Practice Provider	0.76	0.54, 1.06	0.84	0.64, 1.09
Certified Nurse Midwife	0.91	0.49, 1.61	0.56	0.34, 0.91
First Responder (EMT, Paramedic)	1.29	0.70, 2.32	3.71	2.34, 5.93
Nurse	0.43	0.33, 0.55	0.44	0.36, 0.54
Respiratory Therapist	0.97	0.73, 1.30	1.87	1.49, 2.37
Resuscitation experience last 6 months (Ref: 0)				
1–4 times	2.17	1.64, 2.90	1.27	1.05, 1.54
5–10 times	2.30	1.62, 3.27	1.33	1.04, 1.70
>10 times	3.00	2.16, 4.18	1.58	1.25, 2.00
Setting (Ref: Hospital, Neonatal Level III or IV)				
Non-hospital	2.07	1.50, 2.83	1.75	1.36, 2.26
Hospital, No/unknown Neonatal care	1.24	0.82, 1.84	1.23	0.93, 1.62
Hospital, Neonatal Level I or II	1.55	1.23, 1.94	1.01	0.85, 1.20
Didactic Training in laryngeal mask	0.81	0.66, 0.98	1.10	0.95, 1.27
Simulation Training in laryngeal mask	2.62	2.04, 3.40	4.15	3.43, 5.04
Clinical Training in laryngeal mask	8.87	6.90, 11.40	4.74	3.69, 6.10

Abbreviations: aOR: adjusted Odds Ratio; CI: Confidence Interval; EMT: Emergency Medical Technician; NRP: Neonatal Resuscitation Program, Ref: Reference.

1 Confidence includes respondents who answered “very” or “completely” confident.

**Fig. 1:**
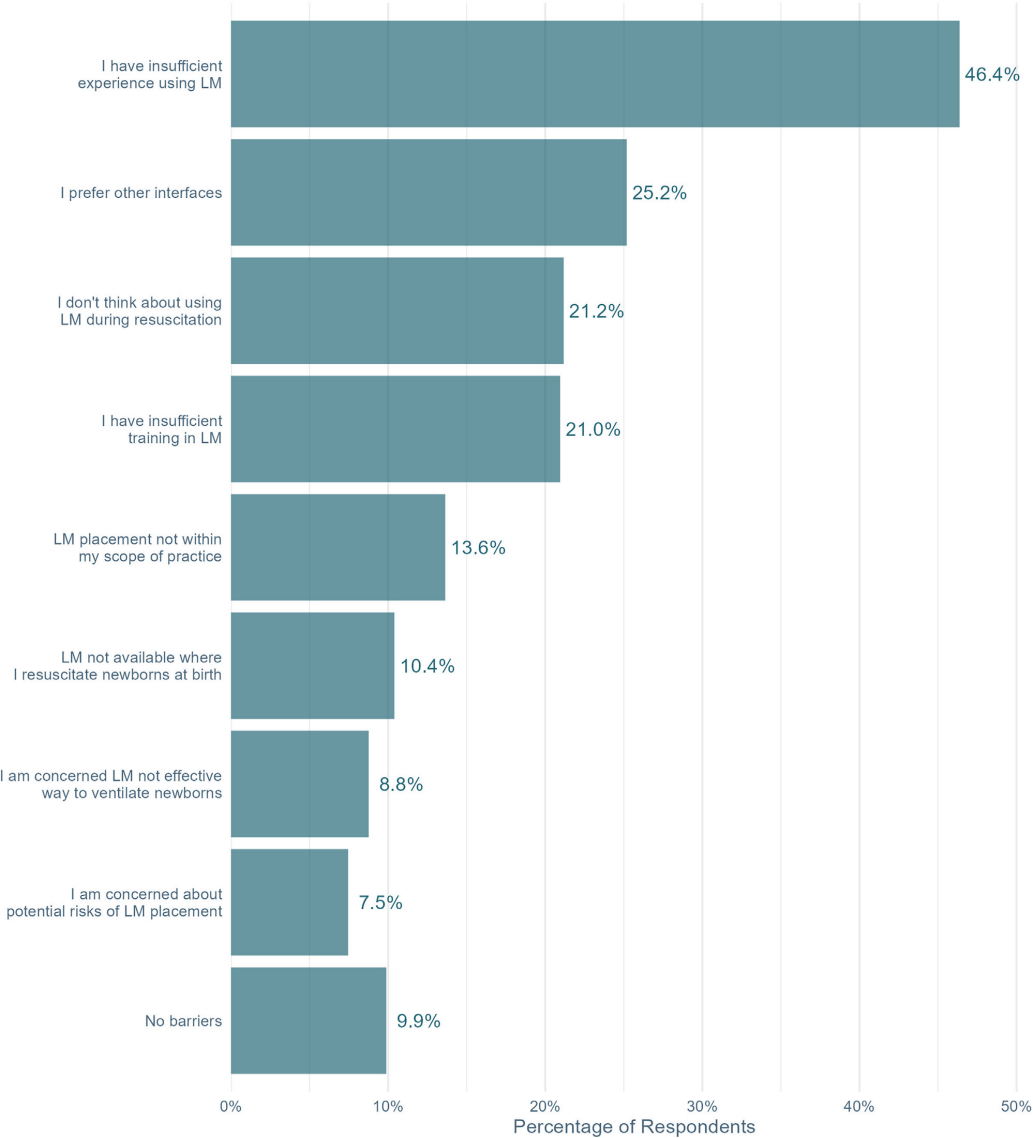
**Barriers to laryngeal mask use identified by NRP Providers and Instructors.** Respondents were asked to identify up to 3 barriers to LM use. Abbreviations: LM: laryngeal mask.

Responses regarding barriers to LM use are presented in Fig. 1. Among respondents, 67% indicated a LM is available in the setting where they resuscitate newborns. When asked to identify the earliest point in the NRP algorithm when LM use is appropriate, 46% of respondents endorsed LM use when facemask PPV is ineffective, and 48% felt the LM to be appropriate when intubation is unsuccessful. Only 3% considered LM use appropriate when PPV is first performed, and 2% indicated LM use is never appropriate for neonatal resuscitation.

Regarding LM implementation questions, 66% of respondents agreed with the statement “I welcome implementation of the laryngeal mask as an alternative airway in my practice setting” (acceptability). Among respondents, 60% agreed that “Implementation of the laryngeal mask as an alternative airway seems like a good match for my practice setting” (appropriateness), and 73% agreed with the statement “Implementation of the laryngeal mask as an alternative airway is possible at my practice setting” (feasibility).

## Discussion

We conducted the first US-based national survey of NRP Providers and Instructors regarding LM use during neonatal resuscitation. Although the NRP has endorsed the LM since 2005,^11^ just 12% of respondents had ever used a LM during resuscitation, and only 25% felt very or completely confident using the LM. While barriers to LM use were identified, most respondents considered implementation of the LM in their practice setting to be acceptable, appropriate, and feasible.

Our results are similar to surveys of neonatal providers in the United Kingdom, Brazil, and North America.^12^, ^13^, ^14^ We found that 67% of respondents have a LM available where they perform newborn resuscitation. Similarly, Goel et al. recently reported that a LM is available in 67% of NICUs and neonatal transport services in Australia and New Zealand.^15^ Ensuring a LM is available in every setting where neonatal resuscitation occurs is a straightforward intervention to support LM implementation.

The most common barriers to LM use endorsed by respondents were insufficient experience, preference for other interfaces, and failure to consider the LM during resuscitation. Our results suggest that hands-on training is likely to increase both use and confidence around the LM for resuscitation. Regarding the latter two barriers identified (preference and consideration), we speculate that many neonatal clinicians prioritize intubation when facemask ventilation fails. However, endotracheal intubation requires advanced technical skills and carries significant procedural risks: almost 50% of intubation procedures require at least 2 attempts, 20% result in adverse events, and 4% are complicated by life-threatening severe adverse events.^5^ In contrast, LM insertion can be learned by inexperienced airway providers in a single training session.^16^, ^17^ Prioritizing LM over intubation during neonatal resuscitation may improve patient safety by establishing effective ventilation quickly, enabling initial resuscitation providers to establish an alternative airway that does not require advanced airway skills, and decreasing the risk of intubation procedural complications.

Most available evidence has focused on LM as the primary interface when PPV is first performed. A recent meta-analysis demonstrated the LM is superior to facemask as the primary PPV interface to avoid ventilation failure for newborns born at ≥34 weeks’ gestation.^18^ Importantly, most trials in that meta-analysis were conducted in low-resource settings. We sought to understand the earliest point of the NRP algorithm when respondents considered LM use to be appropriate in the US. Very few survey respondents considered the LM appropriate as the primary interface. Rather, respondents were evenly divided as to considering LM use appropriate when facemask PPV is ineffective (“can’t ventilate”) or only when intubation is unsuccessful (“can’t ventilate and can’t intubate”).

Our study identified many addressable barriers and may inform implementation strategies for LM use during neonatal resuscitation. At the local level, ensuring a LM is available in every setting where newborn resuscitation is performed is a prerequisite for widespread use. From an educational perspective, hands-on clinical training is likely to improve resuscitation providers’ confidence and use of the LM. Finally, respondents’ perceptions varied regarding when it is appropriate to insert the LM during neonatal resuscitation, suggesting there is opportunity to clarify neonatal resuscitation guidelines on this point.

We acknowledge study limitations. Given the low response rate, responses may not reflect the experience and perceptions of all NRP Providers and Instructors. Nonetheless, the high absolute number (>5,000) of respondents provide confidence in the precision of responses among participants. In addition, the respondents’ professional roles and practice reflect the breadth of providers who are trained in NRP and settings where neonatal resuscitation is performed. Finally, our study is unique in that we also assessed barriers to LM use and implementation readiness.

## Conclusion

Few NRP Providers and Instructors use the LM during neonatal resuscitation. Implementation strategies should address LM availability, educational interventions supporting hands-on clinical training, and outreach promoting the advantages of the LM.

## Funding sources

The DRIVE Network received grant funding from RQI Partners, Chiesi USA, Laerdal Foundation, and Fisher and Paykel. Funders had no role in the study design; in the collection, analysis, or interpretation of data; in the writing of the report; or in the decision to submit the article for publication.

## CRediT authorship contribution statement

**Elizabeth E. Foglia:** Conceptualization, Data curation, Methodology, Writing – original draft. **Birju A. Shah:** Conceptualization, Writing – review & editing. **Lise DeShea:** Conceptualization, Formal analysis, Writing – review & editing. **Kathryn Lander:** Conceptualization, Data curation, Writing – review & editing. **Beena D. Kamath-Rayne:** Conceptualization, Data curation, Writing – review & editing. **Heidi M. Herrick:** Conceptualization, Writing – review & editing. **Jeanette Zaichkin:** Conceptualization, Writing – review & editing. **Sura Lee:** Conceptualization, Writing – review & editing. **Christopher Bonafide:** Conceptualization, Writing – review & editing. **Clara Song:** Conceptualization, Writing – review & editing. **Gene Hallford:** Conceptualization, Writing – review & editing. **Henry C. Lee:** Conceptualization, Writing – review & editing. **Vishal Kapadia:** Conceptualization, Writing – review & editing. **Tina Leone:** Conceptualization, Writing – review & editing. **Justin Josephsen:** Conceptualization, Writing – review & editing. **Arun Gupta:** Conceptualization, Writing – review & editing. **Marya L. Strand:** Conceptualization, Writing – review & editing. **William H. Beasley:** Formal analysis, Writing – review & editing. **Edgardo Szyld:** Conceptualization, Writing – review & editing.

## Declaration of competing interest

The authors declare that they have no known competing financial interests or personal relationships that could have appeared to influence the work reported in this paper.

## Appendix A Supplementary material

Supplementary data 1 Survey Instrument
