# Supplementary material for: Laryngeal mask use during neonatal resuscitation at birth: A United States-based survey of neonatal resuscitation program providers and instructors
Source: Resusc Plus. 2023 Nov 30;17:100515. doi: 10.1016/j.resplu.2023.100515 (PMC10716019; doi:10.1016/j.resplu.2023.100515)
Supplement: Supplementary data 1 — Survey Instrument [file mmc1.docx]

**Supplemental Material:**

**Survey on Laryngeal Mask Use During Neonatal Resuscitation**

Thank you for taking the time to complete this survey. We want to learn about the use of the laryngeal mask (or supraglottic airway) among NRP providers and instructors during resuscitation of newborns immediately after birth.

1. In the past six months, how many times have you participated in the resuscitation of a newborn at birth? Resuscitation includes facemask positive pressure ventilation (PPV) or more extensive interventions.

**O** Never

**O** 1-4 times

**O** 5-10 times

**O** More than 10 times

2. Have you received any training in the placement of a laryngeal mask in a newborn?

**O** Yes *🡪 branching logic to question 2-a*

**O** No

2-a. How was the training provided? Select all that apply:

**O** Didactic training (including lecture, PowerPoint, textbook, video)

**O** Hands-on simulation training on a neonatal manikin

**O** Hands-on clinical training on a newborn

3. Have you ever placed a laryngeal mask during resuscitation of a newborn at birth?

**O** Yes

**O** No

4. How confident are you in placing a laryngeal mask during resuscitation of a newborn at birth?

**O** Not at all confident

**O** Slightly confident

**O** Somewhat confident

**O** Very confident

**O** Completely confident

5. Identify the main barriers (maximum of 3) to your use of a laryngeal mask during resuscitation of a newborn at birth.

**O** I have insufficient training in the laryngeal mask

**O** I have insufficient experience using the laryngeal mask

**O** I prefer other interfaces (such as an endotracheal tube)

**O** Laryngeal mask placement is not within my scope of practice

**O** I am concerned about potential risks of laryngeal mask placement

**O** The laryngeal mask is not available where I resuscitate newborns at birth

**O** I don't think about using the laryngeal mask when I am performing resuscitation at birth

**O** I am concerned the laryngeal mask is not an effective way to ventilate depressed newborns at

birth.

**O** I don't experience any barriers to laryngeal mask use during resuscitation at birth

6. In your practice setting, is a laryngeal mask available in the same room where newborns are resuscitated at birth?

**O** Yes

**O** No

**O** I don't know

7. Please identify the earliest point in the neonatal resuscitation algorithm when you think laryngeal mask insertion is appropriate.

**O** When PPV is first initiated, instead of the facemask

**O** Instead of intubation when facemask PPV is ineffective

**O** When intubation is unsuccessful

**O** Never

The Textbook of Neonatal Resuscitation (8th edition) details ventilation corrective steps to address ineffective face mask ventilation. The step "Alternative Airway" includes insertion of a laryngeal mask or endotracheal tube. However, laryngeal masks are infrequently used in many settings. With that in mind, please respond to the next 3 items about your practice setting for resuscitation of newborns at birth. If you work in more than one practice setting, consider the setting where you spend the most time working.

8. I welcome implementation of the laryngeal mask as an alternative airway in my practice setting.

Completely disagree Disagree Neutral Agree Completely agree

**O O O O O**

9. Implementation of the laryngeal mask as an alternative airway seems like a good match for my practice setting.

Completely disagree Disagree Neutral Agree Completely agree

**O O O O O**

10. Implementation of the laryngeal mask as an alternative airway is possible at my practice setting.

Completely disagree Disagree Neutral Agree Completely agree

**O O O O O**

Demographic Information

11. What is your professional role?

**O** Advanced Practice Provider (NP/PA) *🡪 branching logic to question 11-a*

**O** First Responder (EMT, paramedic)

**O** Certified Nurse Midwife

**O** Nurse

**O** Respiratory Therapist

**O** Physician *🡪 branching logic to questions 11-b and 11-c*

**O** Other: ______

11-a. You answered Advanced Practice Provider. Please specify:

**O** Nurse Practitioner

**O** Physician Assistant

11-b. You answered Physician. Please specify:

**O** Attending

**O** Fellow

**O** Resident

11-c. Field of Medicine (check all that apply)

**O** Emergency Medicine

**O** Family Medicine

**O** General Pediatrics or Hospitalist

**O** Neonatology

**O** Other: ______

12. Please indicate the practice setting where you spend most of your time working

**O** Patient home

**O** Pre-hospital

**O** Birth center

**O** Hospital *🡪 branching logic to questions 12-a and 12-b*

12-a. Indicate the level of neonatal care at the hospital where you spend most of your time working

**O** Level 1

**O** Level 2

**O** Level 3

**O** Level 4

**O** I don't know

**O** Not applicable

12-b. What best describes the hospital where you spend most of your time working?

**O** Teaching hospital

**O** Non-teaching hospital

13. What is your current role in NRP?

**O** Provider

**O** Instructor

When you are done, please click the submit button.
